# 恶性肿瘤合并上腔静脉综合症的治疗进展

**DOI:** 10.3779/j.issn.1009-3419.2016.11.11

**Published:** 2016-11-20

**Authors:** 向征 刘, 诗杰 张, 简 李

**Affiliations:** 100034 北京，北京大学第一医院胸外科 Department of Thoracic Surgery, the First Hospital of Peking University, Beijing 100034, China

**Keywords:** 恶性肿瘤, 上腔静脉综合症, 介入治疗, 血管置换, Malignant tumor, Superior vena cava syndrome, Interventional therapy, Vessel replacement

## Abstract

上腔静脉综合症（superior vena cava syndrome, SVCS）作为胸部恶性肿瘤严重的并发症之一，患者生存期短、生活质量差，大部分采用姑息治疗，随着多学科的发展与治疗技术的进步，越来越多的专家采用各种治疗方式，明显改善了患者的预后，本文对病因表现进行简单归纳，并从内科治疗、介入治疗、放射治疗、外科手术治疗以及质子治疗等几个方面对目前较新的治疗方式进行综述。

## Abstract (en)

Patients with thoracic malignant tumors often suffered from superior vena cava syndrome (SVCS), featured by high morbidity and mortality. Traditionally treated with palliative approaches, SVCS has been widely studied and novel therapeutic approaches have been investigated, focusing on drug therapy, interventional therapy, radiation therapy, surgery and proton therapy. In this manuscript, the progress of therapeutic approaches for SVCS is summarized.

上腔静脉引流人体上半身的血液回流到心脏。上腔静脉综合症（superior vena cava syndrome, SVCS）是指由于通过上腔静脉回流到右心房的血流部分或完全受阻，出现的一组临床综合征^[1]^。

## 1 SVCS的解剖学基础

上腔静脉是人体体循环中重要的组成部分，负责收集来自头臂静脉及奇静脉系统的静脉回流血，于第3胸肋关节下缘注入右心房。颈内静脉收集来自硬脑膜窦、面静脉、舌咽静脉、甲状腺上中静脉等。锁骨下静脉自第1肋外缘由腋静脉延续成，另收集由下颌后静脉后支与耳后静脉，枕静脉回流血液。两侧颈内静脉和锁骨下静脉分别汇合成左右头臂静脉，并于胸锁关节后方汇合。奇静脉自下向上走行，沿途接收食管静脉、肋间后静脉、颈内静脉，于胸7椎体水平与半奇静脉汇合，绕过右肺门，注入上腔静脉。

上腔静脉处在上纵隔一个相对密闭的空间，静脉压相对较低，因此较容易被挤压，且周围被各种器官及大量淋巴结包绕，有胸腺、主支气管、右支气管、主动脉、头臂动脉、肺门及气管旁淋巴结^[1]^。这些组织的病变或形态变化均可以压迫上腔静脉。上腔静脉狭窄导致静脉压上升，远端静脉代偿性扩张，组织水肿、缺氧，局部血液湍流及内皮破坏也可导致血栓形成。

## 2 原发疾病

SVCS最早在1757年被文献报道，原发病是一例梅毒性动脉瘤。直到20世纪中期，梅毒性动脉瘤与结节性纵隔炎仍是文献中报告的大部分SVCS的原发病。随着抗生素的发展应用及对于传染病的有效管理，近年来恶性肿瘤逐渐成为了引起SVCS的最常见因素，其中主要包括肺癌、淋巴瘤、胸腺癌及其他肿瘤^[2, 3]^。除恶性肿瘤外包括纵隔淋巴结结核、静脉血栓、纵隔炎、动脉瘤甚至甲状肿等良性病变，也可以引起SVCS^[4]^。除此之外，随着越来越多的医疗技术的应用，医源性因素也成为不可忽略的原因，例如由心脏起搏器电极脱落或感染等导致的偶发上腔静脉综合征^[5, 6]^。

## 3 症状

### 3.1 静脉回流障碍

SVCS的诊断主要依据临床表现。常见表现为头颈部、上肢的非凹性水肿，部分患者伴口唇及皮肤发绀，症状常在平卧时加重，直立位减轻。根据梗阻部位与奇静脉的关系，可以将SVCS分为两大类：若梗阻部位在奇静脉入口以上者，颈胸部可见静脉怒张，若梗阻部位在奇静脉入口以下者，胸腹壁均可发生曲张。当上腔静脉受压时间较长，还可发生胸部、脊柱、奇静脉、胸阔侧支循环形成，表现出特征性胸壁浅静脉怒张，侧支循环的建立，可以缓解局部的肿胀。部分患者还可出现胸腔及心包积液^[7]^。总体来讲，患者既可以呈现以天为单位的急性起病，也可以存在长达数月的缓慢进展。症状不一定与病情的严重程度正相关，更多取决于自身血流情况与侧支循环情况，有文献^[8]^报道过严重上腔静脉梗阻患者没有出现对应体征的病例。

### 3.2 周围神经器官受损

当气管、食管及喉返神经受压，可引起咳嗽、呼吸困难、进食哽噎感及Horner综合征。急性起病的喉水肿与支气管水肿不但会使患者感到极度不适，也是影响患者生存期的重要因素^[9]^。

### 3.3 中枢神经系统受损

当梗阻时间较长，静脉压升高导致脑部微血管通透性增高，脑组织间隙液体积聚过多，最终导致脑容量增加。脑水肿的临床表现根据发展速度和严重程度各异，重者局灶性体征包括一时性麻痹、半身轻瘫、单或双侧椎体体征等。广泛病变可以引起：头痛、头晕、恶心、呕吐、视神经水肿、血压升高、心动过缓及意识障碍等。若脑内压达到临界值，出现压迫性脑疝，则可表现为中脑或延髓急性压综合征，可出现呕吐头晕、颈强直、角弓反张、意识丧失、呼吸间断甚至停止^[10]^。

## 4 治疗

### 4.1 内科治疗

恶性肿瘤引起的上腔静脉综合征，一般提示肿瘤分期较晚，且症状出现后，患者病情通常进展迅速。内科治疗包括一般支持治疗及化学治疗。一般支持治疗主要包括脱水治疗、激素治疗等。早期一般支持治疗可以改善症状，为进一步治疗提供更好机会，但也有引起血栓、电解质紊乱及水肿加重的可能。因为分化较差的肿瘤有很高比例会引起SVCS，如小细胞肺癌，传统观点认为化学治疗可以有效缓解症状，曾有文献^[3]^报道缓解率达76.9%。化疗还可以配合放疗明显提高治疗效果。对于非小细胞癌患者，单独放疗只有31.3%有效，联合放化疗有效率为63%^[3]^。最近有学者^[11]^报道针对由带有*BRAF*突变的转移性黑色素瘤导致的上腔静脉综合征可以通过服用针对BRAF的靶向药物得到有效缓解。应该指出的是，上腔静脉综合征患者本身即存在血管回流障碍，输液期间必须避免经上肢输入对血管壁有明显刺激作用的液体，很多医院采取下肢静脉或股静脉留置导管输入，可以减轻药物副作用，避免上肢因输液加重的血栓情况及静脉炎。

### 4.2 介入支架治疗

介入支架治疗最早在1986年首次得到尝试，实现了缓解症状的目的。随着影像学和支架材料的发展，支架治疗目前被认为是针对上腔静脉梗阻较为成熟的一种有创检查及治疗手段^[12]^。支架治疗主要针对一般状况差，无法耐受手术或者放疗的患者，或短时间内需要缓解梗阻症状的患者。患者只需要适当监护和局麻处理，平卧在手术台上即可完成治疗^[13]^。与化疗相比介入治疗的优势在于：效果明显，起效快且全身副作用小，可以迅速缓解血管梗阻情况，减轻头颈部及上肢水肿^[14]^。国外文献^[15]^报道，97%-99%的患者在支架介入治疗后症状得到一定程度缓解，头痛通常可以立即缓解，面部水肿在24 h内也可以部分缓解，上肢躯干水肿72 h也会一定程度缓解。最新研究^[16]^显示，覆膜支架比起传统裸支架有效时间更长，12个月通畅率可达94%（裸支架为48%，*P* < 0.05）。但是作为一项有创检查，支架介入治疗同样存在一些问题，一项包含164例样本的研究发现，约12.8%患者介入术后出现并发症，2.4%患者死于并发症，21.9%患者症状复发，最终只有75%的患者得到成功治疗^[17]^。介入治疗的轻微并发症主要包括腹股沟血肿、穿刺部位感染，而严重并发症包括：支架移位、出血、心功能受损、肺栓塞、肺水肿、心包填塞等^[18]^。尽管介入治疗效果明显，专家意见仍不将支架介入治疗纳入对于预期寿命较长患者的一线治疗方案，因为此类患者如果原发病灶得不到处理，症状在几年或几个月后仍有复发的风险。

### 4.3 放射治疗

长久以来，对于大多数SVCS患者，放射治疗作为主要治疗方式的一种得到了重视。尽管近年来支架成为一项可行的姑息治疗方式，但对于大部分患者，放疗仍是重要甚至唯一的治疗方式^[19]^。然而，传统的常规分割放疗技术通常不能完全缓解上腔静脉梗阻，一项研究^[20]^显示，传统放疗后静脉造影显示上腔静脉流量正常的只占11.1%，尸检中只有24.2%患者完全或部分梗阻得到缓解，大约50%-70%患者临床症状有一定缓解。最早由Rubin^[21]^提出的早期大剂量照射（4 Gy/d, 3 d），继以2 Gy/d的剂量进行常规分割照射的治疗方案，因为可以克服局部放射性水肿，从而得到了推广。一项荟萃分析显示，对于小细胞肺癌患者，单独放疗可使77.6%患者的SVCS得到完全缓解，而联合化疗可以使比例升高至83.3%。

正如前文所述，肺癌是SVCS常见病因，近年来，学界对大分割放疗仍存一定争议，大分割放疗的初衷是减少放疗次数加大单次剂量，以达到增加效果、减少并发症的作用，但是近年多项研究^[22, 23]^表明，针对非小细胞肺癌，大分割放疗方案在效果和副作用上与常规方案相比并没有明显差异。此外，立体定向放疗，对于各种类型的肺癌表现出了良好的局部控制率和良好的耐受性^[24, 25]^。现在立体定向放疗已经得到了广泛认可，在增加对于计划靶体积的治疗剂量的同时，由于定位精准，减少了总放射剂量，极大地减轻了对于周围正常组织的损伤^[26]^。一项研究^[27]^显示立体定向放疗对于局部较早期非小细胞肺癌，有效控制率达97%。制定立体定向放疗计划的过程，需要一定时间，这对于需要马上缓解症状的患者并不适用。但是该疗法为体积适当的肿瘤提供了根治性治疗的可能，同时，对于位置合适而对常规放疗不敏感的患者，也可列为治疗选择之一。

### 4.4 质子治疗

质子治疗是近年来出现的一种新兴治疗方式，其原理为通过质子对某些癌症中心组织具有较高的亲和力，对于临近健康组织均有较低的亲和力而对肿瘤细胞产生杀伤作用^[27]^。但是目前缺乏有效的高质量临床证据证明质子在大多数癌症中有显著的作用。另一方面，质子治疗中心和质子治疗本身的高昂成本也限制了质子治疗的推广应用^[28]^。因此在大部分SVCS患者中，质子治疗的获益很小，目前只建议在高度适合的患者身上选择性应用^[29]^。

### 4.5 外科治疗

传统观念将SVCS列为恶性肿瘤手术的禁忌，但随着外科技术的不断发展，越来越多的专家尝试扩大手术切除的适应症。手术已经成为了SVCS不可忽略的治疗手段。根据患者一般情况及病变范围选择手术方式是基本的共识。虽然肿瘤局部分期较晚，决定了总体预后相对较差，但手术可以明显减少患者痛苦，在提高生存质量的同时最大限度延长生存期。早期因为技术有限，外国学者采用姑息性血管转流术来改善局部静脉梗阻情况，但是因为转流血管使用的大隐静脉口径较小且未去除原发病灶，因而预后并不理想，目前转流技术主要用于血管置换术中提供临时性旁路通道，以维持脑供氧，减少手术并发症的发生^[30]^。

传统上肺癌的手术适应证是除Ⅲb期与Ⅳ期外的肺癌，国外有报道^[31-33]^伴SVCS的肺癌患者，平均生存期为3个月-10个月，如果采用姑息性支架治疗，无5年生存，中位生存期为7.9个月，但是采用上腔静脉切除、人造血管重建术后，患者1年、2年、5年生存期分别为70%、25%、12.5%。

与肺癌相比，对于侵袭性胸腺瘤或胸腺癌的手术指征是相对积极的，NCCN 2014年指南已经建议对可以切除的胸腺癌，即使侵犯了重要脏器和大血管，都不应作为手术的绝对禁忌症。但由于胸腺癌恶性程度高，易侵犯心包、大血管及重要脏器，故对手术方式仍存在一定争议。大样本数据显示不完全切除的胸腺癌患者与不行手术，单纯放化疗的患者，5年生存率无统计学差异^[34]^。考虑到胸腺肿瘤相对罕见，目前还是缺乏更有说服力的研究表明手术切除的优势，但是扩大手术切除辅助术后放化疗还是得到了很多外科专家的推荐。

在手术方式方面，目前外科专家常采用的手术切口包括：胸骨正中切口和后外侧切口。一般正中开胸应用较广泛，可在完全暴露手术视野的情况下，确定需扩大切除的范围，血管重建的方式。如肿瘤包裹上腔静脉，在术野有足够空间的情况下，可以先行建立临时或永久的侧支循环。Shields等^[35]^报道，上腔静脉完全阻断45 min是可行的，但考虑不同患者侧支循环情况不同，为了降低脑水肿、脑缺氧发生的可能，绝大多数文献还是支持优先建立转流通路，一般多采用右心耳和左无名静脉间进行转流，然后完成扩大切除及另一侧置换。

对于用于置换的血管材料，一般可以采用自体心包成型、自体大隐静脉、人工血管以及牛静脉等。国外学者^[36]^认为，自体心包是最好的上腔静脉替代物，其优势在于术后不需要长期抗凝治疗，但该方法取材有限，且操作较复杂，目前在临床上尚未得到广泛采用。目前人工血管应用较多见，人工血管具有无需预处理、规格多样、环状壁抗压等优点^[37]^。文献^[38]^报道利用人工血管置换上腔静脉加术后持续抗凝药物治疗使患者获得了优于其他治疗方式的生存获益。

## 5 总结

SVCS作为恶性肿瘤严重的并发症之一，治疗方式一直存在较大争议，随着多学科及仪器技术的进展，从最初的转流手术，到后来的介入治疗、内科治疗、三维放疗、扩大切除人工血管置换等，治疗方式愈发多样化。其中随着研究的深入，手术治疗对提高患者的生存质量、生存期有明显作用，虽然目前各中心研究包含的样本数目较少且对于复杂病例的治疗仍缺乏一定的规范，但可以预见随着样本数目的扩大及相应技术的发展，未来会有更多晚期肿瘤患者能够通过外科手术获益。
